# Revealing bending and force in a soft body through a plant root inspired approach

**DOI:** 10.1038/srep08788

**Published:** 2015-03-05

**Authors:** Chiara Lucarotti, Massimo Totaro, Ali Sadeghi, Barbara Mazzolai, Lucia Beccai

**Affiliations:** 1 Center for Micro-BioRobotics, Istituto Italiano di Tecnologia, Viale Rinaldo Piaggio 34, 56025 Pontedera (Italy); 2 The BioRobotics Institute, Scuola Superiore Sant'Anna, Viale Rinaldo Piaggio 34, 56025 Pontedera (Italy)

## Abstract

An emerging challenge in soft robotics research is to reveal mechanical solicitations in a soft body. Nature provides amazing clues to develop unconventional components that are capable of compliant interactions with the environment and living beings, avoiding mechanical and algorithmic complexity of robotic design. We inspire from plant-root mechanoperception and develop a strategy able to reveal bending and applied force in a soft body with only two sensing elements of the same kind, and a null computational effort. The stretching processes that lead to opposite tissue deformations on the two sides of the root wall are emulated with two tactile sensing elements, made of soft and stretchable materials, which conform to reversible changes in the shape of the body they are built in and follow its deformations. Comparing the two sensory responses, we can discriminate the concave and the convex side of the bent body. Hence, we propose a new strategy to reveal in a soft body the maximum bending angle (or the maximum deflection) and the externally applied force according to the body's mechanical configuration.

Soft robotic approaches point towards a new generation of robots, capable of soft movements, and of soft and safe interaction with environment and humans^1^,^2^,^3^,^4^,^5^,^6^. Compliance is pursued as the key to accomplish delicate and new tasks in the real world, like automation cannot achieve today. In this route, the design of soft sensing systems is one of the main challenges, especially artificial tactile systems that play the major role of providing significant information about the environment where the robot deploys (e.g. interaction forces), as well as its own movements in space (e.g. bending, position).

Different effective strategies for the fabrication of high-performance soft, stretchable and flexible sensing devices have been developed^7^,^8^,^9^,^10^,^11^,^12^,^13^,^14^,^15^,^16^,^17^,^18^,^19^. Soft materials (e.g., elastomers, polymers, fabrics, etc.), with compliances and extensibilities not allowed by rigid components, are investigated today in order to provide diverse functionalities. These include carbon nanotubes embedded in polymeric films for the fabrication of bending^12^ or strain^14^ sensors, stretchable strain sensors based on silver nanowires encased between elastomeric layers^18^, wearable pressure sensors made of ultrathin gold nanowires^7^, resistive pressure sensors built with microstructured elastomers^8^,^13^ or flexible polymer transistors^9^, and flexible or stretchable three-axial force sensors based on conductive textiles^10^ or conductive liquid^19^, respectively. One major issue of these soft sensing approaches, often limiting their applicability, is that sensors respond in a similar manner to different mechanical solicitations - like bending and force^9^. Moreover, though they reach precision and sensibility, they are designed as individual components *per se* instead of built as part of a soft robotic architecture. Therefore, to reveal and discriminate between different mechanical solicitations, like bending and force, the integration of single units, each addressing a specific functionality, is needed, with the result of a dramatic increase in the complexity of robotic design (both mechanical and algorithmic)^2^. This contrasts with the simplifying strategies that characterize many living beings, and that can indeed be considered for endowing robots with new capabilities, pursuing adaptive interactions with unpredictable environments^20^.

We look at plants as living models for new tactile sensing strategies^21^. Essential to its growth and development, a plant adapts to the mechanical stresses (exogeneous) coming from the environment (e.g., wind, soil constraint and mechanical barriers, passing animals etc.) and to those (endogeneous) related to its internal architecture (e.g. turgor pressure driving cell expansion and contributing to plant stability). Plant perception ability, i.e. ‘mechanoperception’, is a key characteristic of all its cells, which deform because of these external and internal mechanical forces^22^. It has been demonstrated how exogenous mechanical stresses due to a touch stimulus induce stretching processes in the epidermal cells at the site of contact (with consequent increase in cytosolic calcium, Ca^2+^, concentration in the epidermis and inner tissues)^23^. In parallel to this, we also learn that when the mature region of the root is manually bent, its cells and tissues are deformed in a different way on the two sides of the root wall. Cells on the convex side are stretched, while cells on the concave side are compressed^22^. Such opposite deformation (positive vs. negative strain) is the natural strategy leading to the significant distinction of triggered internal signal (with an increased Ca^2+^ response) vs. no detectable internal response, for the convex vs. concave walls of the bent root, respectively^23^,^24^. In this investigation, we take inspiration from these aspects of plant-root mechanoperception to design a novel strategy for a soft sensing body (with a constant section) that can discriminate between its convex and concave sides, and that encodes both bending and force, as basic information useful for the future design of new soft perceptive robots.

In the literature very few studies address the integration of soft sensing systems in soft bodies, in particular in bioinspired arms and flexible beams. They detect pressure or are capable of exploiting the movement of the arm to reconstruct their spatial configuration^25^,^26^,^27^,^28^. For octopus-inspired continuum arms, sensorization is achieved with electrotextiles-based force sensors to detect contact^27^, while resistive strain sensors are used to reconstruct the bending direction and the curvature^26^ of the arm^25^. Moreover, deflection sensors based on polyvinylidene fluoride are built for shape tracking of a hyper-flexible beam^28^. However, these systems are not able to distinguish between convex and concave sides and to discriminate between bending and force solicitations, yet they are only capable of a single sensing function (i.e., force or strain sensing).

In this investigation our approach is twofold. First we use soft and flexible materials, like elastomers and conductive textiles, to build two capacitive sensing elements that intimately conform to the shape of the soft body and follow its deformations, inspiring from the stretching processes of plant root tissues. Second, through a bottom-up integrated approach, we look at the combined response of the sensing sites, letting the sensing elements and the body itself act as one single entity rather than different integrated components.

## Results

### Design of the soft sensing body

**Figure 1:**
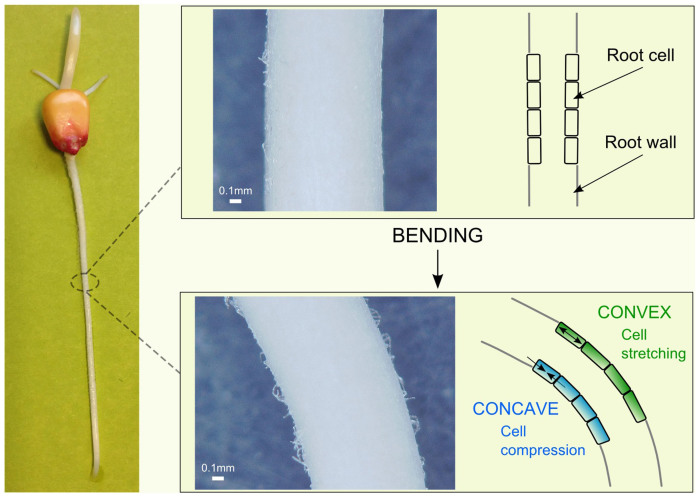
Images and conceptual schematics indicating how manual bending of a plant root causes the deformation of its cells: on the convex side cells are stretched, while on the concave side they are compressed^22^,^23^. On the left side, images of a natural root (*Zea maize*) are shown, with optical microscopy pictures for both rest and bent configurations.

We have retrieved the idea of exploiting the compression and extension of the tissues and cells on the root wall by analysing the biological studies^23^. Hence, we extracted the specification of a cylindrical shape for the soft body, as suitable to mimic the mature zone of the root, i.e. with a constant diameter. In Fig. 1 we exemplify the conceptual idea with the help of images of natural roots.

**Figure 2:**
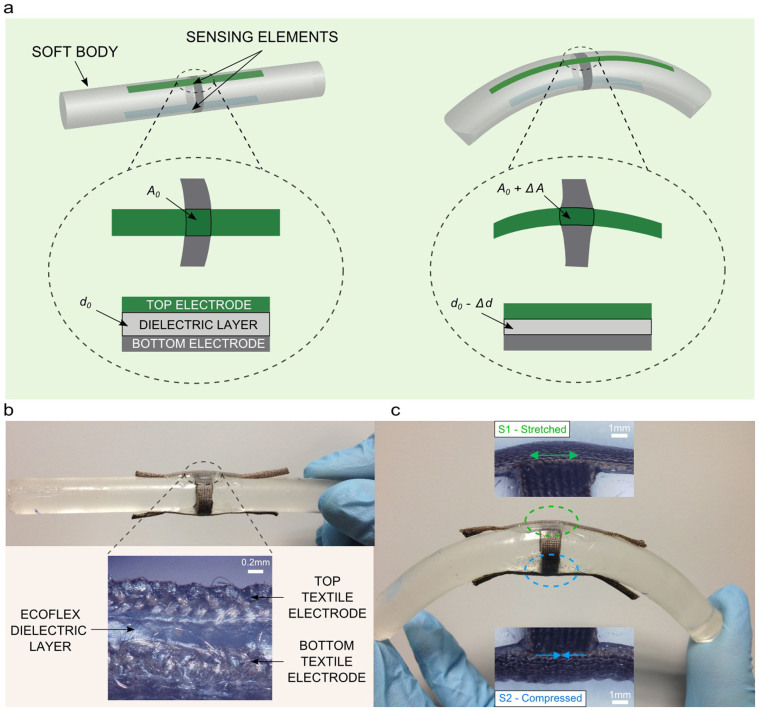
The plant root inspired soft sensing body. (a) Schematics of the artificial soft sensing body with focus on one sensing site, in rest (left) and bending (right) configuration. (b) Picture of the soft sensing body in rest configuration, and inset with the cross-sectional view of one sensing element. (c) Illustration of the bent body, and optical microscopy pictures of the sensing elements on the convex (stretched) and concave (compressed) sides of the PDMS cylinder.

We developed a cylindrical flexible and soft body (having a length *L* of 120 mm and radius *r* of 6 mm), using polydimethylsiloxane (PDMS) since it provides flexibility and softness though acting like a nearly elastic solid. With these dimensions, the PDMS cylindrical body is not deformed by its own gravity^29^ (see Supplementary Information Methods 1), hence gravity effects are not considered in the following analysis. A sensing system is built up into this module including two capacitive sensing elements (namely S1 and S2) at its opposite walls, i.e. at 180° from each other as illustrated in Fig. 2a–c. When the module is bent, opposite convex and concave configurations host the two sensing elements. Such opposite sides of the cylindrical body are subjected to tension and compression solicitation, i.e. on convex and concave respectively. In order to mechanically induce deformation, or change the configuration of the embedded sensing elements, it is crucial that their constituent materials are selected with mechanical characteristics that allow an adaptation to the reversible change in the deformation produced by the bending movement of the soft body. In other words, the key is that S1 and S2 must be part of the body and their mechanical behaviour symbiotic. Hence, both sensing elements are built from two stretchable conductive fabrics parallel electrodes (Electrolycra, Mindsets Ltd), made of a combination of nylon and elastic fibres and plated in silver, separated by a silicone elastomer film (Ecoflex 0010, Smooth-On, 300 μm thick) that serves as a dielectric layer, as illustrated in Fig. 2b–c. The active area of each sensing element is 5 × 5 mm^2^. This is a trade-off between the capability of the electronic system to detect capacitance variations and the hypothesis of having sensing elements positioned in the middle of the soft body length. The selected materials and technology contribute to robust devices through a low cost and easy fabrication process (much faster and simpler than traditional microfabrication techniques). For our purpose, the capacitive transduction mechanism is chosen because of the advantages in terms of higher accuracy, increased sensitivity, long term drift stability, and lower dependency on temperature and humidity, with respect to commonly used piezoresistive, piezoelectric and resistive strain gauge sensing technologies. The variability of the fabrication parameters does not significantly affect performance due to the fact that capacitance variations (instead of the absolute value) are considered. Furthermore, while in this case we present a system with high resolution readout electronics, in general also quite low resolutions could be afforded, since we are mainly interested in distinguishing between different mechanical stimulations. Indeed, this would lead to a further simplification of the whole system.

In this study, we correlate the normalized capacitance variation of the sensing elements S1 and S2 to the different mechanical stimulations applied to the soft body, such as bending and/or an external force, which cause a deformation of all its materials used for both body and sensing. To this purpose, we investigate the response of the system in typical mechanical configurations, like: a cantilever (a) and an eccentrically loaded beam (b), subjected to bending and buckling, respectively; and (c), a beam clamped at both extremities stimulated by both bending and force. In the analysis we consider how the system is constrained and how the force is applied to it, and study its mechanical and electrical behaviour. In this way, from the combined output signal of the sensing elements, the kind of applied solicitation is retrieved and, according to the specific configuration, the bending angle or the maximum deflection of the body itself is revealed.

In the literature, soft tissues are analysed by means of non-linear models (i.e. Moonley-Rivlin, Ogden, or Fung^30^,^31^). However, in this case we are interested on the strain of the body surface, and as shown below, it is always less or around 0.1. Indeed, the classical theory of elasticity, usually applied to rigid bodies, gives reliable predictions as discussed in the following analysis.

To model the strain induced by a bending stimulus, the main hypothesis is that, when the system is mechanically stressed, the capacitive sensing element is subjected to the same deformation of the body surface, since it conforms to the structure. Such deformation results in a change both in the thickness of the dielectric layer and in the sensing area *A*_0_, with a consequent variation in the capacitance value. Several assumptions are made (see Supplementary Information Methods 2) and the resulting fractional change in capacitance Δ*C*/*C*_0_, due to the induced strain ε, is given by
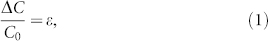
where *C*_0_ is the nominal capacitance in case of absent stimulation.

### Cantilever beam configuration

In this case, we consider the soft sensing body as a cantilever beam subjected to bending, and correlate the capacitance variation of the sensing elements to the maximum bending angle. Our study is supported by a theoretical model (see Supplementary Information Methods 3a).

**Figure 3:**
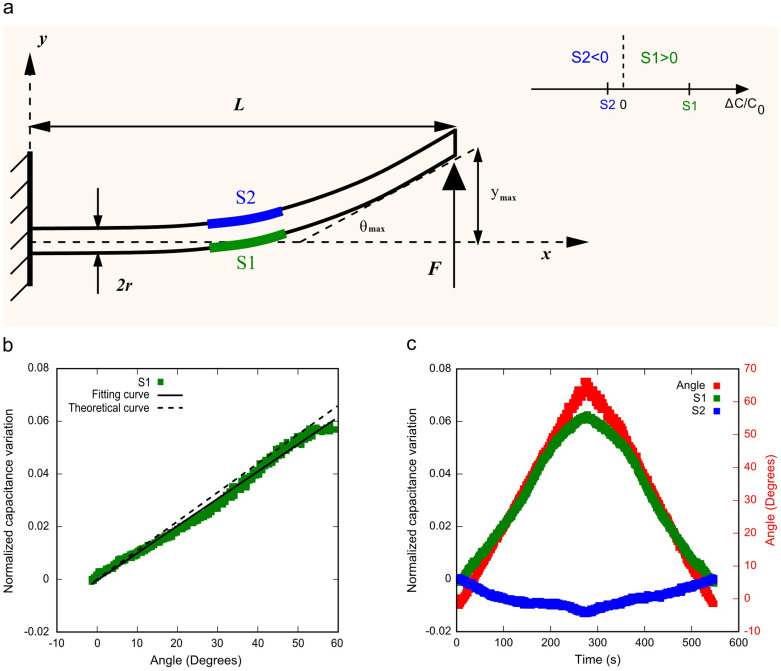
Cantilever beam configuration. (a) Schematic of a cantilever beam with a concentrated force *F* at the free extremity, and the sensing elements S1 and S2 positioned at the centre of the PDMS body. (b) Characteristics (normalized capacitance variation vs. angle) of the sensing convex side (S1) when a bending is produced. Experimental (green squares), fitting (solid line) and theoretical (dashed line) curves are compared. Since angles are represented in degrees, the factor π/180 has been introduced in Eq. (2) for depicting the theoretical curve. (c) Time responses of S1 (green squares) and S2 (blue squares), located on the convex and concave sides, respectively, together with the bending angle (red squares) measured with the 2-axis accelerometer during a bending/unbending cycle. (See also Supplementary Data D2- Fig. D3, D9a).

A cantilever beam, with length *L* and radius *r* clamped at one extremity, and free to move at the other one, is considered. Then, a force *F* is applied at the free extremity, while the sensing elements S1 and S2 are positioned at the centre of the beam, as shown in Fig. 3a. For S1, which is at the convex side, the correlation between the normalized change in the capacitance value Δ*C*/*C*_0_ and the maximum bending angle *θ_max_* is positive, and it results
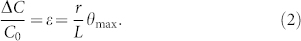
On the other hand, for S2 that is at the concave side, the strain is negative. Therefore, the relative capacitance variation should be also negative with the same absolute value of the one corresponding to S1. However, the hypothesis that the mechanical deformations of the sensing elements conform to those of the underlying beam surface is strictly valid only for positive strains. Indeed, in the case of negative strain the materials are compressed. Due to the fact that the thickness of the sensing element is comparable to (or even larger of) the beam curvature, such compression causes mechanical deformations (such as a wrinkling of the different constituent layers) which do not conform to the beam surface (for more details, see Supplementary Information Methods 3a). The result is that Eq. (2) cannot be applied to the compressed sensing element S2. However, the whole capacitance variation is still negative, even if it is quite less in amplitude than the one relative to S1, as shown in Fig. 3c. This fact allows us to distinguish the concave and the convex side of the bent body and to quantitatively evaluate the strain (thereby the bending angle) by exploiting the sensing element that is being stretched.

Figures 3b–c present the experimental results in the case the soft sensing body is considered as a cantilever beam subjected to bending due to an externally applied force at the free end. The output signal (normalized capacitance variation vs. angle) of S1 at the convex side is illustrated in Fig. 3b. The sensing element responds to an increase in the bending angle with an increase in the normalized capacitance variation, with a good agreement between the experimental data and the theoretical predictions. In fact, the slope of the curve is about 10^−3^ with an error of 6.5% with respect to the theoretical model. The response of the sensing system and the angle measurement versus time are depicted in Fig. 3c. S1 outputs a positive capacitance variation, while S2 is characterized by a negative capacitance change, with much smaller amplitude with respect to the one of S1. Therefore these experimental data confirm that the concave and the convex side of the bent body can be clearly distinguished. Also, the maximum bending angle can be measured, correlating it to the positive strain of S1. In this case, as shown in Fig. 3b, the S1 response is linear in the range 0°–60°, with null hysteresis for bending cycles (for more details, see Supplementary Information Data D1, Fig. D1). Finally, the minimum detectable deflection angle is 0.025 rad (~1.5°) that is due to the minimum capacitance variation detectable with the electronic circuitry.

### Eccentrically loaded beam

In the case of an eccentrically loaded beam, we study the behaviour of the soft sensing body when subjected to buckling due to a compressive load, and correlate the nominal change in the capacitance value to the maximum deflection of the beam.

**Figure 4:**
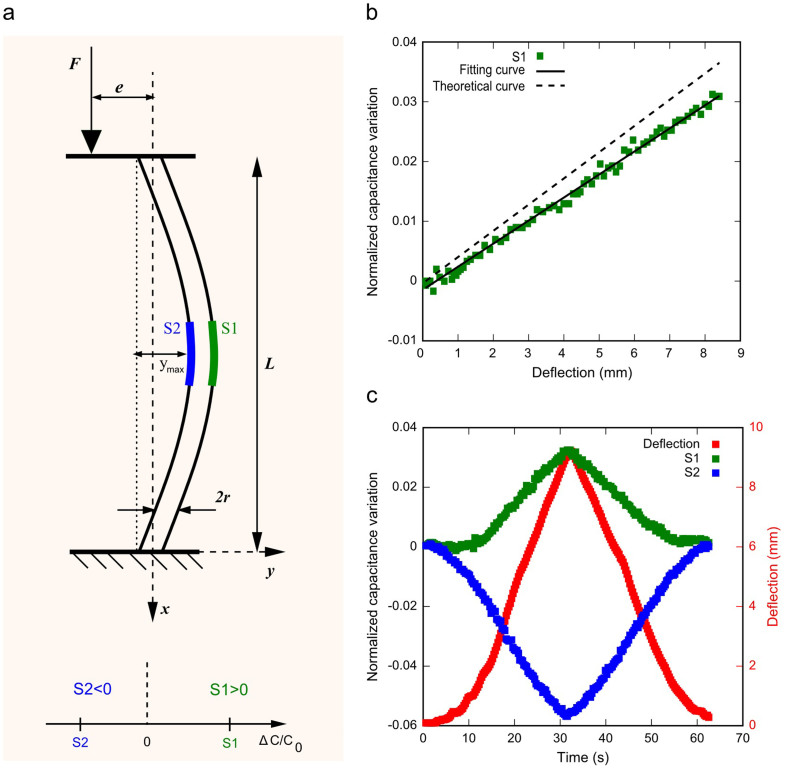
Eccentrically loaded beam configuration. (a) Schematic representing the buckling of an eccentrically loaded beam with eccentricity *e* between the beam vertical axis and the application point of a force *F*, and with the sensing elements S1 and S2 positioned at the centre of the body. (b) Characteristics (normalized capacitance variation vs. deflection) of the sensing convex side (S1) when the beam is subjected to buckling. Experimental (green squares), fitting (solid line) and theoretical (dashed line) curves are compared. (c) Time responses of the sensing elements located on the convex (S1) and concave (S2) side, respectively, of the soft body during a load/unload cycle. The graphs show the normalized capacitance variation of S1 (green squares) and S2 (blue squares), together with the effective deflection (red squares) measured by means of the laser displacement sensor. (See also Supplementary Data D2- Fig. D4, D9b).

Consider a beam with length *L* and radius *r* clamped at both extremities, with an eccentricity *e* between the beam vertical axis and the application point of the compressive force *F*, and the sensing elements S1 and S2 embedded at the beam centre (see Fig. 4a). For small deflections, the correlation between the normalized capacitance variation and the maximum deflection *y_max_* (see Supplementary Information Methods 3b) results
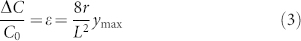
As we can observe, the strain ε, and consequently the normalized capacitance variation, do not depend on the eccentricity *e*, but only on the geometrical dimensions of the beam. Also in this case, like in the previous, the relation is verified only for the stretched sensing element (S1) at the convex side. In the case of compression, the element should experience a negative strain with the same absolute amplitude, but (as we can see in Fig. 4c) this is not observed. Nevertheless, the capacitance variation of the compressed sensing element (S2) at the concave side is negative, allowing us to distinguish the convex and the concave side of the bent body, and to determine the maximum deflection by applying Eq. (3) to the stretched sensing element.

The characteristic curve (normalized capacitance variation vs. deflection) of S1 when the body is subjected to buckling is depicted in Fig. 4b: an increase in the normalized change in the capacitance value corresponds to an increase in the deflection of the beam, with a general consistency between the experimental data and the theoretical assumptions (the slope of the curve is about 4.4 × 10^−3^ with an error of 13% with respect to Eq. (3)). Figure 4c shows the response of the sensing body and the maximum deflection of the beam over time when the system is subjected to buckling: according to the theoretical analysis, S1 on the convex side and S2 on the concave side, respond with a positive and a negative change of capacitance, respectively. In this case as shown in Fig. 4b, the S1 response is linear in the deflection range of 0–8.3 mm. This corresponds to a deflection range of about 7% of the soft body length (of 120 mm). The minimum detectable deflection, due to the electronic read-out capabilities, is 0.4 mm. Moreover, the transfer curve does not show hysteresis in load-unload cycles (for more details, see Supplementary Information Data D1, Fig. D2).

### Beam clamped at both extremities

Starting from the theoretical investigation (see Supplementary Information Methods 3c), we analyse the behaviour of the soft body clamped at both ends when subjected to a bending solicitation and to an externally applied force. Comparing S1 and S2 output signals, we can establish in which side the external force is applied, also providing its value, and the maximum deflection of the beam.

**Figure 5:**
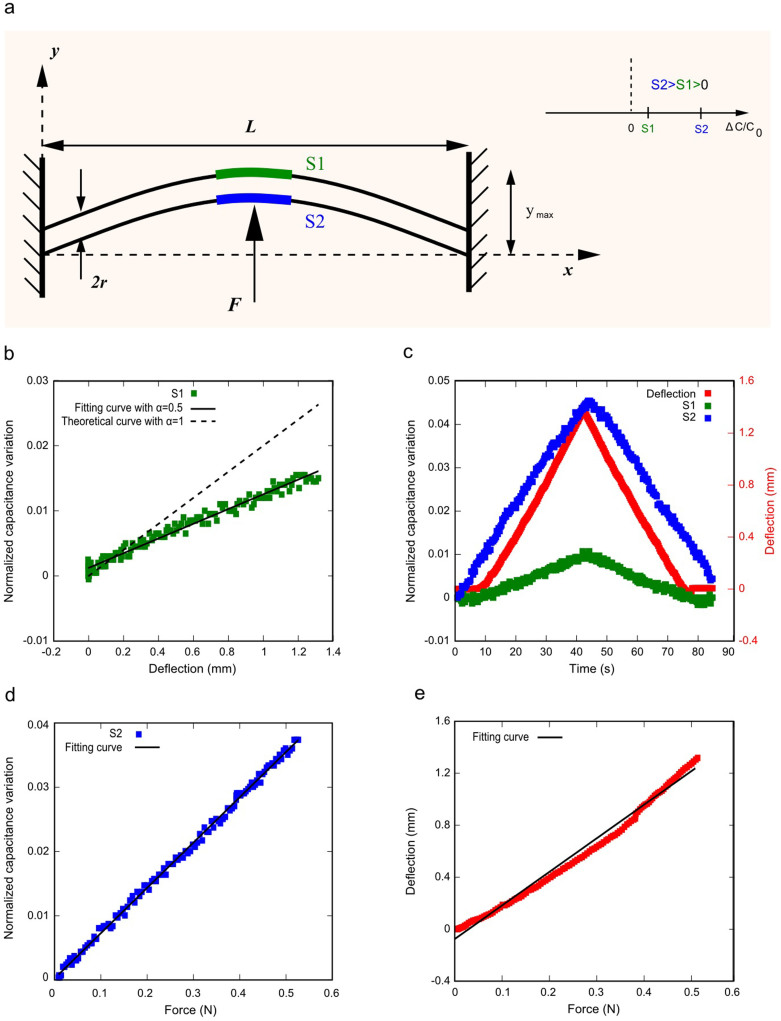
Configuration of a beam clamped at both extremities subjected to both bending and force solicitations. (a) Schematic showing a beam clamped at both extremities with an externally applied force *F* at the middle and with the sensing elements S1 and S2 at the beam centre. (b) Characteristics (normalized capacitance variation vs. deflection) of the sensing convex side (S1). Experimental (green squares), fitting (solid line) and theoretical (dashed line) curves are compared. (c) Time responses of the soft sensing elements when an external force is applied in the middle of the body. Graphs show: the normalized capacitance variations of the sensing concave side (S2) in contact with the intending probe (blue squares) and of the sensing convex wall (S1) (green squares), together with the effective deflection (red squares). (d) Characteristics (normalized capacitance variation vs. force) of the sensing concave side (S2). (e) Force/deflection characteristics of the PDMS soft sensing body. (See also Supplementary Data D2- Fig. D5, D9c).

In the case of a beam, with length *L* and radius *r*, clamped at both extremities and indented with a force *F* at its middle region (where S1 and S2 are positioned, see Fig. 5a), the correlation between the normalized capacitance variation and the maximum deflection *y_max_*, is
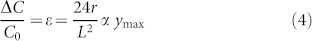
where α is an experimental fitting factor, as explained below.

The proposed equation is valid for positive strain, which in this case occurs at the convex side, where S1 is located. Otherwise, at the concave side, where S2 is, a small negative capacitance variation should be observed in the case of pure beam buckling. However, in this case also an external indentation force, having direction perpendicular to the soft body side, is applied directly on the area where S2 is embedded. Supposing that this force mainly varies the thickness of the dielectric layer *d*_0_, the resulting normalized capacitance variation corresponds to (see Supplementary Information Methods 2)
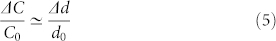
We know that a force so applied also causes the bending of the soft structure, with the consequent variation in the area of the capacitive element therein embedded. As discussed in the first case (cantilever beam), for a sensing element on the concave side, we can expect this contribution to be negative, with its relative amplitude at most comparable with the case of pure bending of the body (the strain is in the same order of magnitude). However, we can observe that *L* is typically in the order of centimetres (or even more), while *d*_0_ in our case is about 300 μm. Then, the quantity in Eq. (5) is much larger than that in Eq. (4), and thus it represents a predominant contribution of the external force stimulus. This allows us to distinguish the concave and the convex side of the bent body, to quantitatively determine the applied force (exploiting the sensing element characterization), and to evaluate the maximum deflection by means of Eq. (4) applied to the sensing element on the convex side, S1. Finally, concerning the maximum deflection, we need to introduce an experimental fitting factor α in Eq. (4). Indeed, one of the hypotheses is that the beam bends in such a way that its cross-section is constant for any *x* value: this is verified for a rigid material body (see Supplementary Information Methods 3c), with a consequent value of α = 1. Otherwise, in the case of a soft beam, the pressure applied by a rigid probe can relevantly deform the beam shape in the region around the application point, where the sensing elements are positioned. The result is that the effective strain on the sensing element, and thereby its capacitance variation, can be different with respect to the theoretical prediction. However, as shown in experimental results, also in the case of a beam made of soft material like PDMS, the relation is linear. Thus, to correlate the effective deflection to the capacitance variation, a corrective experimental fitting factor can be introduced. Moreover, the amplitude of the signal detected by the pressed sensing element is still much larger than that of the sensor positioned on the opposite side. Then, all previous consideration made about the discrimination of bending and external force solicitations are still valid.

Figure 5b presents the output characteristics (normalized capacitance variation vs. deflection) of the sensing element S1 on the convex side of the body when both bending and force stimulations are applied. As shown in the graph, the sensing element on the concave side responds to an increase in the deflection with an increasing normalized capacitance variation. However, the deviation between the experimental data and the theoretical model for a rigid body (i.e. α = 1) is significant. As explained above, this is due to the fact that our analysis is strictly valid for a body made of rigid materials. Since the response is linear up to 1.32 mm (with negligible hysteresis for load/unload cycles; see Supplementary Information Data D1, Fig. D3), we can fit from the experimental curve the corrective factor, which in our case is around 0.5. For example, in the case of a beam made of a rigid but elastic core (i.e., a metallic spring) with a soft coating (i.e., rubber) the value of α is much closer to the unit (see Supplementary Information Data D2, Fig. D4). However, even with a soft material beam, the behaviour can be reconstructed correctly, since the capacitance variations of S1 and S2 are still both positive, as shown in the time response of Fig. 5c. In the case of a PDMS beam, the force range detected by S2 is 0–0.52 N, as shown in Fig. 5d, for a maximum deflection of 1.32 mm, as depicted in Fig. 5e. It should be pointed out that in the previous case of buckling column, the S1 behaviour is governed by Eq. (3) (depicted in dashed line in Fig. 4b), while in this case we should consider Eq. (4) (shown in dashed line in Fig. 5b) for S1. Then, according to the mechanical configuration considered, the same normalized capacitance variation can correspond to different deflection quantities.

Finally, in this configuration, the minimum detectable deflection measured by S1 is around 70 μm, while the resolution of measured applied force (detected by S2) can be extracted from the experimental curve of Fig. 5d, and it corresponds to around 10 mN. We have to note that the detectable force range depends on the constituent materials of the soft sensing body. For example, in the case of the rigid but elastic core with the soft rubber coating, the same maximum deflection of 1.32 mm is obtained with larger forces, i.e. up to 3.27 N (see Supplementary Information Data D2, Fig. D5).

## Discussion

We present a novel soft sensing strategy by imitating the opposite mechanical behaviour of tissues at the concave and convex walls of a plant root. In an artificial soft sensing body we obtain two major results: (1) the discrimination of the convex and concave sides of the bent structure; and (2) the detection of different mechanical stimulations, like bending and force.

The investigated sensing body consists of a soft flexible PDMS cylinder with two built-in capacitive sensing elements. Their constituent materials are characterized by mechanical features that allow their adaptation to reversible changes in the shape of the cylinder. The result of both material's choice and design is a soft body with inherent tactile sensing, all operating as one single system.

In our study, by using the plant-root inspired approach, we demonstrate that it is possible to provide information about the interaction forces with the environment and the movement of a soft body. The outputs of this study are relevant for the emerging field of soft robotics^5^, where the potential applications depend on a trade-off between compliance and accuracy of soft bodies performing specific tasks. In this context, tactile sensory information is useful to implement a final desired behaviour. Importantly, we address one of the major issues of soft sensing technology. In fact, different mechanical solicitations – e.g. external force and bending - affect similarly the same soft sensor architecture^9^,^32^ since the physical mechanisms underlying transduction are akin, and they are principally based on the deformation behaviour of the used materials. Indeed, even if they have characteristics of flexibility, softness and stretchability that allow their integration in 3D systems, soft sensors need to respond properly, in function of the applied mechanical input. Therefore, the design of the sensors should be changed and complicated (e.g. to this aim in electronic skins dielectric materials need microstructuring^33^), or an array of same sensors should be used in combination with *ad-hoc* signal processing algorithms. In contrast, we compare responses of two opposite (i.e. concave and convex) sensing sites built by a simple and low cost technology.

We focus on typical mechanical configurations and estimate the mechanical and electrical behaviour of the system, supporting it with a theoretical analysis. The results validate the theoretical predictions in each scenario, by depicting how the information about the bending angle or the maximum deflection of the body can be retrieved, and eventually how to discriminate between different mechanical stimulations applied contemporarily, i.e. bending and external force.

It is worth to emphasize that the proposed strategy is trivial from the computational point of view. Specifically, given a particular mechanical configuration, we retrieve the applied stimulation by looking at the sign of the responses of the sensing elements S1 and S2. In the cantilever beam configuration, the capacitance variation of the concave sensing side is negative, while the response on the convex side is positive. In addition, the latter can be used to quantitatively determine the maximum bending angle of the cantilever. In the case of an eccentrically loaded beam, the concave side presents a negative capacitance variation, while in the convex side it is positive, and it is also exploited to reveal the maximum deflection of the body. Finally when the body is clamped at both extremities, both responses present a positive variation. However, the signal on the concave side is always much larger in amplitude (due to force applied directly on it) with respect to the convex side. This allows to distinguish the two sides, and, as demonstrated above, to measure simultaneously the externally applied force and the maximum deflection of the beam. In this last case, since our model is strictly valid for an ideal rigid body, we can introduce an experimental corrective factor in the equation that correlates the capacitance variation with the maximum deflection, in order to retrieve the correct value also in the case of soft modules, as shown in the experimental results. Moreover, experimental data highlight that the response time of the soft sensing body is not limited by hysteresis effects (see Supplementary Data D1, Fig. D1–D3).

Therefore, from a simple comparison between the two sensing signals, the applied solicitation is retrieved and, according to the specific configuration, the maximum bending angle or the maximum deflection of the body can be determined.

The strategy to distinguish between two opposite sides of the soft bent structure holds potential to elaborate new ways in determining the shape of a soft robot, and, in general, due to its null computational cost, it could be applied in many scenarios. In particular, today innovative robotic systems inspired by plant roots, able to move autonomously and explore the environment efficiently and in a non-destructive way, are being studied opening new paths for soil monitoring, and exploration of the environment^21^,^34^. In the natural system, mechanoperception plays a key role since the root uses efficient strategies to circumnavigate the barriers and to direct its growth^22^ and, in doing this, it implements bending movements. Hence, a bioinspired artificial root should be able to perceive barriers to growth but at the same time its own movements, like bending. Moreover, the plant root tissues adapt and conform to the soil or environment. The imitation of all this aspects can be investigated through soft robotic approaches where the computation required for the control is aimed to be outsourced to the body of the robot (i.e. morphological computation approach^35^).

A potential application to mention is represented by wearable devices for health monitoring^36^, where human motion could be detected together with contact forces by integrating the two soft sensing systems at the opposite sides of the considered body part (e.g. ankle, foot, knee, etc.). This would represent an advancement with respect to stretchable electronic materials attached to the body that can measure only strain^32^. Another possible field of application is represented by soft surgical tools (e.g. probes, endoscopes, etc.), which are more flexible and compliant^37^, and have sensing capability, thus safer because they are able to smartly adapt to human bodies. More in general, cases in which a careful yet sensory guided handling or manipulation of fragile elements, or interaction with humans is needed would benefit from applying the proposed approach.

It is noteworthy that, in spite the technological embodiment we used to validate the presented strategy results well suited and robust, also different materials could be employed (e.g. CNTs^32^,^38^,^39^, graphene^40^,^41^, PEDOT:stretchable PSS^42^,^43^, nanostructured elastomers^44^,^45^ for the electrodes of the capacitor), opening new applications for current stretchable electronics and tactile systems. This is true as long as the conformability and soft characteristics (of both body and sensing) are kept, while allowing correct and robust transduction.

In conclusion, we believe that through the presented bioinspired sensing approach, a significant new step forward is made for the development of inbuilt soft body and sensing overcoming drawbacks of conventional sensors' integration, and paving the way to new perspectives for future generations of soft robots and related technologies, mainly for areas of wearable systems, medicine and rehabilitation.

## Methods

### Fabrication process and implementation of the soft sensing body

The flexible and soft body consists of a polydimethylsiloxane (PDMS) cylinder (having length of 120 mm and diameter of 12 mm), obtained by curing a mixed and degassed PDMS prepolymer (Dow Corning, Sylgard 184, with a ratio of base to cross-linker of 10:1 by mass) for 48 hours in a Delrin mould. In parallel to the flexible module the sensing elements S1 and S2 are prepared as follows. Electrolycra (Mindsets Online, Middlesex University, UK) stretchable conductive textile (made of weaved Silver/Nylon elastic fibres) is cut by CO_2_ laser (VLS 3.50, Universal Laser Systems, Inc., USA) and employed for both the top and the bottom electrodes (5 mm × 5 mm, 700 μm thick), while Ecoflex silicone elastomer (Smooth-On 0010, USA), with dielectric constant *ε_r_* = 2.5, is selected as material for the dielectric layer due to its mechanical properties that allow the conformability to the deformation of the body. In particular, the silicone elastomer is spin-coated from a 1:1 (weight/weight) solution of Ecoflex – Part A and Ecoflex – Part B at 300 rpm for 30 s, then cured for 5 hours at room temperature to produce a film with thickness of 360 μm. The Ecoflex substrate is cut into 10 mm × 10 mm rectangle (to allow the perfect gluing to the body and to avoid short circuits between the top and the bottom electrodes), and placed in between the electrodes. Uncured Ecoflex silicone rubber is employed to attach the electrodes to the dielectric layer. Consequently, the capacitive sensing elements are embedded at the central part of the soft and elastic cylindrical structure, at 180° from each other. This step is achieved by using uncured Ecoflex silicone rubber and the curing is performed at room temperature for 4 hours. Finally, around the central region of the cylindrical structure a thin layer of Ecoflex (around 100 μm thick) is formed by dispensing a small quantity of material.

### Capacitance readout electronics

The capacitance readout electronics consists of two 24 bits capacitance-to-digital converters (AD7747, Analog Device Inc., Nordwood, MA, USA), assembled on a custom Printed Circuit Board (PCB). To reduce the effect of parasitic capacitances a differential configuration is implemented, with a dummy capacitance as reference. Moreover, each sensing element is connected to the PCB by means of shielded coaxial cables. The resolution of the converter is 1 fF and, due to the strategies described above, the RMS noise is reduced to about 1.3 fF. Thus, the minimum detectable signal is about 4 fF (three times larger than RMS noise). Since 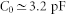, the minimum detectable normalized capacitance variation is 1.25 × 10^−3^. Finally, from the converter, digital data are elaborated by a 32-bit PIC (PIC32MX460F512L, Microchip Technology Inc., Chandler, AZ, USA) microcontroller and then transmitted to a PC by means of USB communication.

### Experimental setup and protocol

Before starting the measurements, the nominal capacitance of each element of the sensing system is evaluated by means of a precision LCR Meter (EA980A, Agilent Technologies Inc., Palo Alto, CA, USA). Three experimental setups are developed (see Supplementary Data D3, Fig. D6–D9) in order to reproduce the selected mechanical configurations (i.e., a cantilever (a) and eccentrically loaded (b) beam subjected to bending and buckling, respectively, and beam clamped at both extremities (c) subjected to both externally applied force and bending), and to evaluate the output response of the sensing body. In all three configurations we consider as S1 the sensing element positioned on the convex side of the bent body, while as S2 the sensing element on the concave side.

In case (a), a mechanical support is used to clamp the soft body at one extremity in order to apply a bending simulation (therefore a force at the free end of the body). The free end is connected through an inextensible Nylon wire to the load cell of electromechanical equipment (Instron 4464, Instron Corporation, Canton, MA, USA). The bending is performed by applying a vertical tension to the wire with a velocity of 10 mm/min and an acquisition frequency of 10 Hz. Finally, a linear capacitive accelerometer (LIS2L02AL, STMicroelectronics Inc., Geneva, CH) is fixed to the soft structure free end to measure the maximum angle deflection (as a function of the time) while the system is bent.

Configuration (b) corresponds to buckling of the soft body due to a compressive load with an eccentricity between the beam vertical axis and the load application point. The beam is clamped at both extremities by means of two Plexiglas supports (cut by laser) with holes housing both extremities of the body. A vertical compressive force (providing a bending movement of the beam) is applied by means of the same electromechanical equipment used in the previous configuration, with a constant velocity of 10 mm/min and an acquisition frequency of 10 Hz. A laser displacement sensor (optoNCDT 1401, MICRO-EPSILON, Ortenburg, Germany), aligned to the sensing element S2, is employed to record (with an acquisition frequency of 10 Hz) the deflection of the beam (with a micrometre scale accuracy) due to bending.

The third experimental setup is built to experiment configuration (c). Specifically, the beam is clamped at both extremities by means of the same mechanical support used in case (b), and a force is applied at the middle of the beam perpendicularly to its surface where S2 is embedded. First, a Delrin probe with flat head (8 mm × 8 mm) is aligned to the sensing area of the sensing element S2 by means of three orthogonal manual micrometric translational stages with crossed roller bearing (M-105.10, PI, Karlsruhe, Germany); then, the precise positioning of the loading probe in the normal direction is obtained by means of a servo-controlled micrometric translational stage (M-111.1, PI, Karlsruhe, Germany). At its opposite side, the probe is mechanically interfaced to a 6-components load cell (ATI NANO 17 SI-25-0.25, Apex, NC, USA) capable of recording the indentation force applied to the soft sensing body (with acquisition frequency of 10 Hz). The probe advanced towards the sensing area of the sensing element S2 at a constant velocity of 0.05 mm/s, gradually increasing the displacement and the applied load. The abovementioned laser displacement sensor, aligned to the top electrode of the sensing element S1, is integrated in the experimental setup in order to record (with acquisition frequency of 10 Hz) the effective deflection of the sensing system due to the indentation force.

In all the experimental sessions, the electrodes of each sensing element are connected to the readout electronics by means of shielded coaxial cables in order to measure the capacitance variations of the capacitors. The readout electronics is connected to a PC via USB where an ad-hoc Graphical User Interface (GUI) is used to acquire, in a synchronized way: the two capacitance values; the angle (when needed, by means of the accelerometer); and, the force (when needed, by means of the load cell). Moreover, when required, the laser displacement sensor is connected to the same PC in order to record the effective deflection of the sensing system over time.

## Author Contributions

C.L. and M.T. contributed equally to this work. C.L. and L.B. initiated this work and with M.T. designed the experiments. C.L., M.T. and A.S. built the experimental setup. C.L. and M.T. performed the experiments. M.T. developed the model. C.L., M.T. and L.B. analyzed the data, discussed and wrote the manuscript. L.B. and B.M. oversaw and advised the research.

## Supplementary Material

Supplementary Information Supplementary Information

## References

[b1] Iida F. & Laschi C. Soft robotics: challenges and perspectives. Procedia Comput Sci 7, 99–102, 10.1016/j.procs.2011.12.030 (2011).

[b2] Kim S., Laschi C. & Trimmer B. Soft robotics: a bioinspired evolution in robotics. Trends Biotechnol 31, 287–294, 10.1016/j.tibtech.2013.03.002 (2013). 23582470

[b3] Pfeifer R., Lungarella M. & Iida F. The challenges ahead for bio-inspired ‘soft’ robotics. Commun. ACM 55, 76–87, 10.1145/2366316.2366335 (2012).

[b4] Trivedi D., Rahn C. D., Kier W. M. & Walker I. D. Soft robotics: Biological inspiration, state of the art, and future research. Appl Bionics Biomech 5, 99–117, 10.1080/11762320802557865 (2008).

[b5] Trimmer B. Soft robots. Curr Biol 23, R639–R641, 10.1016/j.cub.2013.04.070 (2013). 23928077

[b6] Lipson H. Challenges and opportunities for design, simulation, and fabrication of soft robots. SoRo 1, 21–27 (2013).

[b7] Shu G. *et al.* A wearable and highly sensitive pressure sensor with ultrathin gold nanowires. Nat Commun 5, 10.1038/ncomms4132 (2014). 24495897

[b8] Pan L. *et al.* An ultra-sensitive resistive pressure sensor based on hollow-sphere microstructure induced elasticity in conducting polymer film. Nat Commun 5, 10.1038/ncomms4002 (2014). 24389734

[b9] Schwartz G. *et al.* Flexible polymer transistors with high pressure sensitivity for application in electronic skin and health monitoring. Nat Commun 4, 1859, 10.1038/ncomms2832 (2013). 23673644

[b10] Viry L. *et al.* Flexible three-axial force sensor for soft and highly sensitive artificial touch. Adv Mater 26, 2659–2664, 10.1002/adma.201305064 (2014). 24677245 PMC4264044

[b11] Majidi C., Kramer R. & Wood R. J. A non-differential elastomer curvature sensor for softer-than-skin electronics. Smart Mater Struct 20, 105017 (2011).

[b12] Wichmann M. H. G., Buschhorn S. T., Böger L., Adelung R. & Schulte K. Direction sensitive bending sensors based on multi-wall carbon nanotube/epoxy nanocomposites. Nanotechnology 19, 475503 (2008). 21836274 10.1088/0957-4484/19/47/475503

[b13] Mannsfeld S. C. B. *et al.* Highly sensitive flexible pressure sensors with microstructured rubber dielectric layers. Nat Mater 9, 859–864, 10.1038/nmat2834 (2010). 20835231

[b14] Lipomi D. J. *et al.* Skin-like pressure and strain sensors based on transparent elastic films of carbon nanotubes. Nat Nano 6, 788–792, 10.1038/nnano.2011.184 (2011). 22020121

[b15] Yun S. *et al.* Polymer-waveguide-based flexible tactile sensor array for dynamic response. Adv Mater 26, 4474–4480, 10.1002/adma.201305850 (2014). 24711161

[b16] Kong J.-H., Jang N.-S., Kim S.-H. & Kim J.-M. Simple and rapid micropatterning of conductive carbon composites and its application to elastic strain sensors. Carbon 77, 199–207, 10.1016/j.carbon.2014.05.022 (2014).

[b17] Yan C. *et al.* Highly stretchable piezoresistive graphene–nanocellulose nanopaper for strain sensors. Adv Mater 26, 2022–2027, 10.1002/adma.201304742 (2014). 24343930

[b18] Amjadi M., Pichitpajongkit A., Lee S., Ryu S. & Park I. Highly stretchable and sensitive strain sensor based on silver nanowire–elastomer nanocomposite. ACS Nano 8, 5154–5163, 10.1021/nn501204t (2014). 24749972

[b19] Noda K., Matsumoto K. & Shimoyama I. Stretchable tri-axis force sensor using conductive liquid. Sensors Actuat A-Phys 215, 123–129, 10.1016/j.sna.2013.09.031 (2014).

[b20] Trimmer B. A journal of soft robotics: why now? SoRo 1, 1–4 (2013).

[b21] Sadeghi A., Tonazzini A., Popova L. & Mazzolai B. A novel growing device inspired by plant root soil penetration behaviors. PLoS ONE 9, e90139, 10.1371/journal.pone.0090139 (2014). 24587244 PMC3934970

[b22] Monshausen G. B. & Haswell E. S. A force of nature: molecular mechanisms of mechanoperception in plants. J Exp Bot 64, 4663–4680, 10.1093/jxb/ert204 (2013). 23913953 PMC3817949

[b23] Monshausen G. B., Bibikova T. N., Weisenseel M. H. & Gilroy S. Ca2+ regulates reactive oxygen species production and pH during mechanosensing in Arabidopsis roots. Plant Cell 21, 2341–2356, 10.1105/tpc.109.068395 (2009). 19654264 PMC2751959

[b24] Monshausen G. B. & Gilroy S. Feeling green: mechanosensing in plants. Trends Cell Biol 19, 228–235, 10.1016/j.tcb.2009.02.005 (2009). 19342240

[b25] Cianchetti M. *et al.* Design and development of a soft robotic octopus arm exploiting embodied intelligence. Paper presented at ICRA-2012 conference, St. Paul. (5 14 2012).

[b26] Cianchetti M. *et al.* Sensorization of continuum soft robots for reconstructing their spatial configuration. Paper presented at BioRob-2012 conference, Rome. (6 24 2012).

[b27] Jinping H., Bonser R. H. & Jeronimidis G. Developing sensorized arm skin for an octopus inspired robot. Paper presented at ICRA-2012 conference, St. Paul. (5 14 2012).

[b28] Shapiro Y., Kosa G. & Wolf A. Shape tracking of planar hyper-flexible beams via embedded PVDF deflection sensors. IEEE/ASME Trans. Mechatronics 19, 1260–1267, 10.1109/tmech.2013.2278251 (2014).

[b29] Cox S. J. & McCarthy C. M. The Shape of the Tallest Column. SIAM J Math Anal 29, 547–554, 10.1137/S0036141097314537 (1998).

[b30] Holzapfel G. A. Nonlinear solid mechanics: A continuum approach for engineering. (John Wiley and Sons, Ltd., 2004).

[b31] Fung Y. C. Biomechanics: Mechanical properties of living tissues. (Springer, 1993).

[b32] Yamada T. *et al.* A stretchable carbon nanotube strain sensor for human-motion detection. Nat Nano 6, 296–301, 10.1038/nnano.2011.36 (2011). 21441912

[b33] Sekitani T., Zschieschang U., Klauk H. & Someya T. Flexible organic transistors and circuits with extreme bending stability. Nat Mater 9, 1015–1022, 10.1038/nmat2896 (2010). 21057499

[b34] Mazzolai B., Beccai L. & Mattoli V. Plants as model in biomimetics and biorobotics: New perspectives. Front Bioeng Biotechnol 2, 10.3389/fbioe.2014.00002 (2014). PMC4126448 25152878

[b35] Pfeifer R. & Bongard J. C. How the body shapes the way we think: a new view of intelligence (The MIT Press, 2006).

[b36] Ya-Li Z. *et al.* Unobtrusive sensing and wearable devices for health informatics. IEEE Trans Biomed Eng 61, 1538–1554, 10.1109/tbme.2014.2309951 (2014). 24759283 PMC7176476

[b37] Kang B. *et al.* Towards accurate robot-assisted neuroendoscopy using an ergonomic handling interface and a lightweight robot. Paper presented at EMBC-2014 conference, Chicago. (8 26 2014). 10.1109/EMBC.2014.6945208 25571576

[b38] Cao Q. & Rogers J. A. Ultrathin films of single-walled carbon nanotubes for electronics and sensors: a review of fundamental and applied aspects. Adv Mater 21, 29–53, 10.1002/adma.200801995 (2009).

[b39] Cai L. *et al.* Super-stretchable, transparent carbon nanotube-based capacitive strain sensors for human motion detection. Sci Rep 3, 10.1038/srep03048 (2013). PMC6505716 24157842

[b40] Cheng Q. *et al.* Graphene and nanostructured MnO2 composite electrodes for supercapacitors. Carbon 49, 2917–2925, 10.1016/j.carbon.2011.02.068 (2011).

[b41] Wang D.-W. *et al.* Fabrication of graphene/polyaniline composite paper via in situ anodic electropolymerization for high-performance flexible electrode. ACS Nano 3, 1745–1752, 10.1021/nn900297m (2009). 19489559

[b42] Vosgueritchian M., Lipomi D. J. & Bao Z. Highly conductive and transparent PEDOT:PSS films with a fluorosurfactant for stretchable and flexible transparent electrodes. Adv Funct Mater 22, 421–428, 10.1002/adfm.201101775 (2012).

[b43] Greco F. *et al.* Ultra-thin conductive free-standing PEDOT/PSS nanofilms. Soft Matter 7, 10642–10650, 10.1039/c1sm06174g (2011).

[b44] Corbelli G., Ghisleri C., Marelli M., Milani P. & Ravagnan L. Highly deformable nanostructured elastomeric electrodes with improving conductivity upon cyclical stretching. Adv Mater 23, 4504–4508, 10.1002/adma.201102463 (2011). 21997303

[b45] Rosset S., Niklaus M., Dubois P. & Shea H. R. Metal ion implantation for the fabrication of stretchable electrodes on elastomers. Adv Funct Mater 19, 470–478, 10.1002/adfm.200801218 (2009).

