# Supplementary material for: Revealing bending and force in a soft body through a plant root inspired approach
Source: Sci Rep. 2015 Mar 5;5:8788. doi: 10.1038/srep08788 (PMC4350075; doi:10.1038/srep08788)
Supplement: Supplementary Information [file srep08788-s1.pdf]

Revealing bending and force in a soft body through a plant root inspired approach

Chiara Lucarotti[1,2], Massimo Totaro[1], Ali Sadeghi[1], Barbara Mazzolai[1],

Lucia Beccai[1*]

[1]Center for Micro-BioRobotics @SSSA, Istituto Italiano di Tecnologia, Viale Rinaldo Piaggio 34, 56025 Pontedera (Italy)

[2]The Biorobotics Institute, Scuola Superiore Sant'Anna, Viale Rinaldo Piaggio 34, 56025 Pontedera (Italy)

[*] corresponding author:

Lucia Beccai

Center for Micro-BioRobotics, Istituto Italiano di Tecnologia

Viale Rinaldo Piaggio 34, 56025 Pontedera (PI), Italy

phone: +39 050 883079/400; fax: +39 050 883101

email: lucia.beccai@iit.it

Supplementary Methods 1

The polydimethylsiloxane (PDMS) cylindrical body is not deformed by its own gravity. Indeed, the maximum height of a cylindrical column with density ρ and Young's modulus E standing under its own weight is[29] $h_{crit} = \sqrt[3]{\frac{9}{16\rho g} B^2 r^2 EI}$, where g is the gravity acceleration, I the second moment of area, and $B \approx 1.8663$ is the least positive root of the Bessel function of order -1/3. In the case of PDMS, ρ = 965 kg/m^3 and E = 1.8MPa. Moreover, the cylindrical body has $r = 6$ mm, and it results $h_{crit} \approx 24$ cm. Therefore, given that the length L of the soft body is 12 cm, gravity effects are not considered in our analysis.

Supplementary Methods 2

From an electrical point of view, the nominal capacitance of the single sensing element can be considered as the capacitance of two parallel electrode plates, as explained in equation (M1)

$$C_0 = k_0 \frac{A_0}{d_0} \tag{M1}$$

where k_0, A_0 and d_0 are the permittivity, the sensing area and the dielectric thickness, respectively. A natural logarithm is applied to equation (M1) to transform the product into a sum and the quotient into a subtraction, respectively, as follows

$$ln(C_0) = ln(k_0) + ln(A_0) - ln(d_0) \tag{M2}$$

Differentiating, it results in

$$\frac{C_0'}{C_0} = \frac{k_0'}{k_0} + \frac{A_0'}{A_0} - \frac{d_0'}{d_0} \tag{M3}$$

that can be approximated at the first order as

$$\frac{\Delta C}{C_0} = \frac{\Delta k}{k_0} + \frac{\Delta A}{A_0} - \frac{\Delta d}{d_0} \tag{M4}$$

The term $\Delta A/A_0$ represents the fractional change in the area due to the strain ε. The area is the product of the width w and the length l of the top electrode, then

$$\frac{\Delta A}{A_0} = \frac{\Delta l}{l_0} + \frac{\Delta w}{w_0} = \varepsilon - \nu\varepsilon = (1 - \nu)\varepsilon\,, \tag{M5}$$

since $\Delta l/l_0 = \varepsilon$, and $\Delta w/w_0 = -\nu\varepsilon$, with ν the Poisson's ratio coefficient. Moreover,

$$\frac{\Delta d}{d_0} = -\nu\varepsilon \tag{M6}$$

Then, the resulting fractional change in capacitance is represented by

$$\frac{\Delta C}{C_0} = \frac{\Delta k}{k_0} + (1 - \nu)\varepsilon + \nu\varepsilon = \varepsilon\,, \tag{M7}$$

since we can consider that the permittivity k_0 does not vary when the strain ε is applied.

On the other hand, when an external force is applied, as discussed in the main text, the predominant effect is due to the change in the distance between the two electrodes, resulting

$$\Delta C = k_0 \frac{A_0}{d_0 - \Delta d} - k_0 \frac{A_0}{d_0} = k_0 \frac{A_0}{d_0} \frac{\Delta d}{d_0 - \Delta d} \tag{M8}$$

If $d_0 \gg \Delta d$, then

$$\frac{\Delta C}{C_0} = \frac{\Delta d}{d_0 - \Delta d} \cong \frac{\Delta d}{d_0} \tag{M9}$$

Supplementary Methods 3

A theoretical model is presented in order to describe the mechanical and electrical behaviour of the soft sensing body when subjected to bending and/or force stimulations. In particular, some configurations of the body, representing typical mechanical stimulations are analysed; these being: (a) a cantilever and (b) an

eccentrically loaded beam subjected to bending and buckling, respectively; (c) beam clamped at both extremities subjected to both force and bending.

3a. Bending of a cantilever beam

In this configuration, the body is subjected to a bending solicitation, and the aim is to correlate the capacitance variation of the sensing elements to the maximum bending angle.

From a mechanical point of view, a cantilever beam (as presented in Fig. 2a) with length L and radius r is clamped at one extremity and free to move at the other one; a force F is applied at the beam free extremity, while the sensing elements S1 and S2 are positioned at the centre of the beam. The resulting deflection of the cantilever beam $y(x)$ in the y- direction is given by

$$y(x) = \frac{Fx^2}{6EI}(3L - x) \qquad \text{(M10)}$$

where E is the Young's modulus and I the moment of inertia. The slope $\theta(x)$ is determined from the first derivative of the deflection and is given by

$$\theta(x) = \frac{dy(x)}{dx} = \frac{F}{2EI}(2Lx - x^2) \qquad \text{(M11)}$$

Therefore, the maximum deflection y_{max} is represented by

$$y_{max} = y(L) = \frac{FL^3}{3EI} \qquad \text{(M12)}$$

and the maximum slope θ_{max} is given by

$$\theta_{max} = \theta(L) = \frac{FL^2}{2EI} \qquad \text{(M13)}$$

It is possible to assume that the predominant effect is represented by the uniaxial strain ε, given by

$$\varepsilon = -h\frac{\partial^2 y(x)}{\partial x^2} = -h\frac{F}{EI}(L - x)\,, \tag{M14}$$

where h is the vertical coordinate of the considered surface with respect to the neutral axis. We can note that below the neutral axis the beam is stretched (and we have a positive strain), while above it is compressed (with a resulting negative strain). The y-axis, defining the deflection, is taken positive upward. Then, if the sensing element is positioned at the centre of the beam and on the lower surface, we have to consider $x = L/2$ and $h = -r$. The resulting strain ε is positive and it is given by equation (M15)

$$\varepsilon = \frac{F}{EI}\frac{L}{2}r = \frac{r}{L}\theta_{max} \tag{M15}$$

As previously explained in equation (M7), the nominal capacitance variation corresponds to the strain ε to which the system is subjected. Therefore, the correlation between the capacitance variation and the bending angle results

$$\frac{\Delta C}{C_0} = \varepsilon = \frac{r}{L}\theta_{max} \tag{M16}$$

If angles are measured in degree, we have to introduce the factor π/180 and the slope of equation (M16) is given by

$$s = \frac{\pi}{180}\frac{r}{L} \tag{M17}$$

As we can note from equation (M14), in the case of a sensing element positioned on the upper surface of the beam ($h = r$), the strain should be negative, and so the relative capacitance variation, with the same absolute value of the sensing element considered above (positioned on the opposite surface). However, the assumption that the sensing element follows the mechanical deformations of the beam surface is true only for positive strains, since the sensing element constituent materials can stretch conformably to the substrate. On the other hand, in the case of negative strain, the

materials are subjected to compression. By considering the thickness of the sensing element comparable (or even larger) to the beam curvature, abovementioned compression phenomenon results in mechanical deformations (i.e., wrinkles of the different constituent layers, as depicted in Fig.2a) which do not conform to the body surface. Therefore, from these considerations, we can understand that equation (M16) cannot be applied to a compressed sensing element.

3b. Buckling of an eccentrically loaded beam

In this configuration, we want to describe the behaviour of the soft sensing body when subjected to buckling due to a compressive load, and to correlate the nominal capacitance variation to the maximum deflection of the beam. In the general case of axial compressive load, the buckling of a beam occurs when the critical load $F_{crit} = \pi^2 EI \,/L^2$ is reached. However, in many situations, the load is not perfectly axial, and an eccentricity e between the load application point and the beam vertical axis is present. In this latter case, the buckling occurs even for very small compressive loads. Consider a beam with length L and radius r clamped at both extremities, with an eccentricity e between the beam vertical axis and the force F application point, and the sensing elements S1 and S2 positioned at the beam centre (as depicted in Fig. 3a): the deflection $y(x)$ of the beam in the y- direction is given by

$$y(x) = \; e\left(\tan\frac{kL}{2}\; \sin kx + \cos kx - 1\right) \tag{M18}$$

where $k = \sqrt{F/EI}$. The maximum deflection y_{max} occurs at $x = L/2$ as explained in equation (M19)

$$y_{max} = y(L/2) = e\left(\sec\frac{kL}{2} - 1\right) \tag{M19}$$

The strain ε on the stretched surface of the beam ($h = r$) is given by

$$\varepsilon = -h\frac{\partial y(x)^2}{\partial^2 x} = r\ e\ k^2\left(\tan\frac{kL}{2}\sin kx + \cos kx\right) \tag{M20}$$

and for $x = L/2$,

$$\varepsilon_{max} = r\ e\ k^2 \sec\frac{kL}{2} \tag{M21}$$

which relates the maximum strain ε_{max} to the compressive load F (since $k = \sqrt{F/EI}$). By combining equations (M19) and (M21), the relation between the maximum strain and the maximum deflection can be obtained

$$\varepsilon_{max} = \frac{4r}{L^2}(y_{max} + e)\,\mathrm{acos}^2\left(\frac{e}{y_{max} + e}\right) \tag{M22}$$

From an electrical point of view, the normalized capacitance variation can be correlated to the maximum strain, as explained in the following equation

$$\frac{\Delta C}{C_0} = \varepsilon_{max} = r\ e\ k^2 \sec\frac{kL}{2} \tag{M23}$$

or, alternatively, by considering the maximum deflection y_{max}, we can correlate the normalized capacitance variation to the compressive load, as described in equation (M24)

$$\frac{\Delta C}{C_0} = \frac{4r}{L^2}(y_{max} + e)\,\mathrm{acos}^2\left(\frac{e}{y_{max} + e}\right) \tag{M24}$$

Equation (M24) can also be written as follows

$$\frac{\Delta C}{C_0} = f(x) = \frac{4r}{L^2}e(x+1)\,\mathrm{acos}^2\left(\frac{1}{x+1}\right) \tag{M25}$$

where $x = y_{max}/e$. Then, expanding the function at the first order around $x = 0$ as a Taylor's series, it is possible to obtain

$$f(x) \simeq f(0) + f'(x)|_{x=0} \cdot x = \tag{M26}$$

$$= 0 + \frac{4r}{L^2} e \left[1 \cdot \mathrm{acos}^2 \left(\frac{1}{x+1} \right) \Big|_{x=0} \right.$$

$$\left. + (x+1)|_{x=0} \lim_{x \to 0} \frac{2\,\mathrm{acos}\left(\frac{1}{1+x}\right)}{(x+1)\sqrt{x(x+2)}} \right] x =$$

$$= \frac{8r}{L^2} e \cdot x$$

since, using de l'Hôpital's rule, we can find

$$\lim_{x \to 0} \frac{2\,\mathrm{acos}\left(\frac{1}{1+x}\right)}{(x+1)\sqrt{x(x+2)}} \tag{M27}$$

$$= 2 \lim_{x \to 0} \left(\frac{1}{(x+1)\sqrt{x(x+2)}} \cdot \frac{\sqrt{x(x+2)}}{2x^2 + 4x + 1} \right)$$

$$= 2$$

The result obtained in equation (M27) is valid for $x \ll 1$, which means that $y_{max} \ll e$. Finally, it is possible to obtain

$$\frac{\Delta C}{C_0} = \frac{8r}{L^2} e \cdot x = \frac{8r}{L^2} y_{max} \tag{M28}$$

In the last case, we can observe that the strain, and consequently the capacitance variation, does not depend anymore on the eccentricity e, but only on the geometrical dimensions of the beam. Also in this case, the above relations are verified only for a stretched sensing element.

3c. Bending of a beam clamped at both extremities

In this case, we want to describe the behaviour of a body clamped at both ends when subjected to both bending and force solicitation. The combination of S1 and S2 signals will let us establish which side is subjected to force, giving also its value, and the maximum deflection of the beam.

Consider a beam with length L and radius r clamped at both extremities (see Fig. 4a): the deflection $y(x)$ of the beam, under a concentrated force F in the middle of the beam (where the sensing elements S1 and S2 are positioned) and in the y-direction, for $0 < x < L/2$, is given by equation (M29)

$$y(x) = \frac{Fx^2}{48EI}(3L - 4x) \tag{M29}$$

The maximum deflection y_{max} is represented by

$$y_{max} = y(L/2) = \frac{FL^3}{192EI} \tag{M30}$$

As previously, the predominant contribute is due to the uniaxial strain ε given by

$$\varepsilon = -h\frac{\partial^2 y(x)}{\partial x^2} = -h\frac{F}{8EI}(L - 4x) \tag{M31}$$

In this case, the strain is positive above the neutral axis. Therefore, when the sensing element is positioned at the centre of the beam ($x = L/2$) and on the upper surface ($h = r$ in our reference system), combining equations (M30) and (M31) the strain is given by

$$\varepsilon = \frac{FLr}{8EI} = \frac{24r}{L^2}y_{max} \tag{M32}$$

From an electrical point of view, the nominal capacitance variation of the sensing element may be considered the same as previously explained in equation (M1), and the fractional change in capacitance is the same of equation (M7). Therefore,

$$\frac{\Delta C}{C_0} = \varepsilon = \frac{24r}{L^2}y_{max} \tag{M33}$$

As already observed in the case of the cantilever beam, the model is valid for positive strain, which in this case occurs on the upper surface. Equation (M33) is valid in the case of a beam made of rigid materials, as explained in the main text. To consider the

deviation observed in the case of soft materials, we should introduce a corrective factor α in equation (M33), obtaining

$$\frac{\Delta C}{C_0} = \varepsilon = \frac{24r}{L^2} \alpha \, y_{max} \qquad \text{(M34)}$$

In the case of a beam with a rigid but elastic core (i.e., a metallic spring) and a soft coating (i.e., rubber), the deviation to equation (M33) is very small, and we have $\alpha \simeq 1$, as shown in the section below. Otherwise, for body entirely made of a deformable material, such as PDMS, the corrective factor can differ significantly to 1 (in our experiments we found a value around 0.5).

Finally, for a sensing element positioned on the lower surface, a small negative capacitance variation should be observed in the case of pure beam buckling. However, in this case, a force is applied directly on it. As discussed in the main text, the force mainly varies the thickness of the sensing element d_0, therefore the resulting normalized capacitance variation corresponds to equation (M8).

Supplementary Data D1

In the following, we show the performance of our soft sensing body in terms of hysteresis.

In the case of a cantilever beam, we demonstrate that the response (normalized capacitance variation vs. angle) of the sensing element S1 is linear in the range 0°-60° with no hysteresis (as shown in Fig. D1).

Figure D2 depicts the transfer curve (normalized capacitance variation vs. deflection) of the sensing convex side S1 in the configuration of an eccentrically loaded beam: the sensor response is linear in the deflection range of 0-8.3 mm, and it does not show hysteresis.

Finally, in the case of a beam clamped at both extremities, the S1 response (normalized capacitance variation vs. deflection) is linear up to a deflection of 1.32 mm, and the hysteresis is negligible, as shown in Fig. D3.

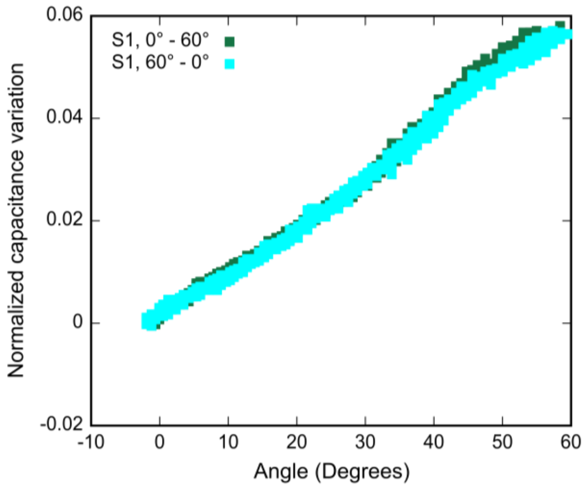

Supplementary Figure D1. Characteristics (normalized capacitance variation vs. angle) of the sensing convex side S1 in the cantilever configuration for a bending/unbending cycle in the range 0°-60°.

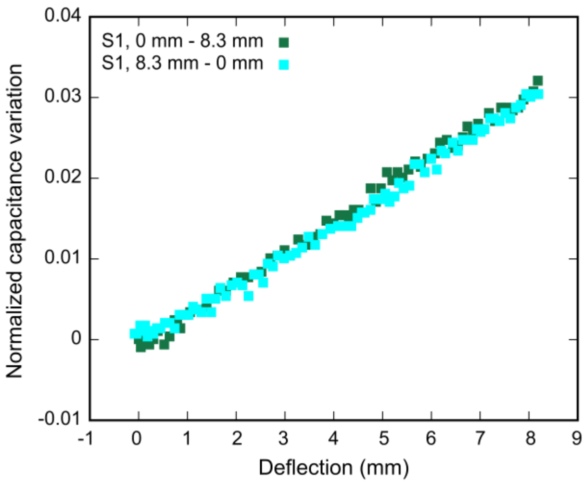

Supplementary Figure D2. Characteristics (normalized capacitance variation vs. deflection) of the sensing convex side S1 under a load/unload cycle in the eccentrically loaded beam configuration.

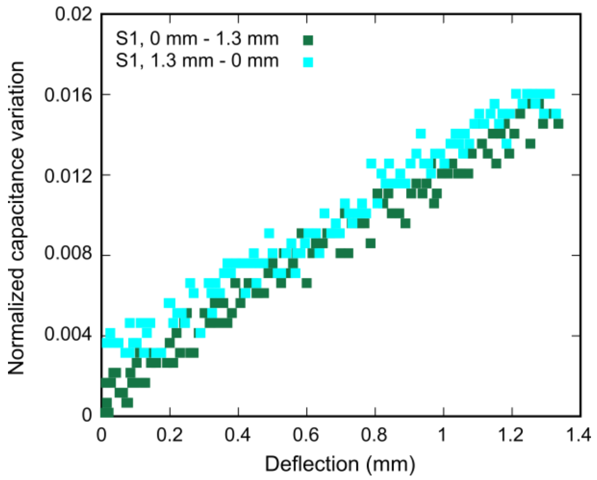

Supplementary Figure D3. Characteristics (normalized capacitance variation vs. deflection) of the sensing convex side S1 for a beam clamped at both ends when the system is subjected to both bending and force solicitations.

Supplementary Data D2

As shown above, in the case of a beam clamped at both ends, we need to introduce a corrective factor to equation (M33) for soft material bodies. To investigate the correctness of equation (M33), we made a module (with length of 120 mm and radius of 6 mm) composed of a metallic spring with a rubber coating. The length L and the radius r are the same of the PDMS body presented in the main text. In the same way, two stretchable capacitive sensors are placed at centre of the beam at 180° each other. The characteristic of the sensing element S1 on the convex side of the body is shown in Fig. D4. We can note that the experimental data are consistent with the theoretical curve (dashed black line) of equation (M33). In particular, in this case we obtain $\alpha \simeq 1$, much closer to the unit with respect to the PDMS module.

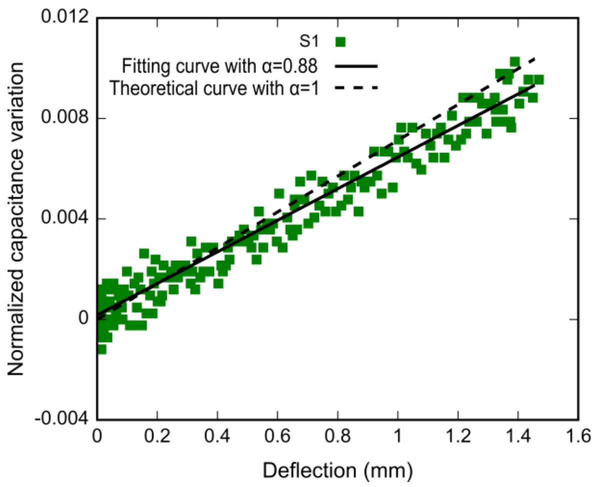

Supplementary Figure D4. Characteristics (normalized capacitance variation vs. deflection) of the sensing element S1 on the convex side for a module made of a metallic spring with a rubber coating, in the configuration of a beam clamped at both extremities subjected to both bending and force solicitations.

Moreover, in such less-deformable module the deflection of 1.32 mm is obtained with larger forces (i.e., 3.27 N, as depicted in Fig. D5) with respect to the case of the PDMS beam, where a force of about 0.55 N is enough to reach the same deflection. This demonstrates, as expected, that the force/deflection characteristic depends on the mechanical properties of the body in which the sensing elements are embedded.

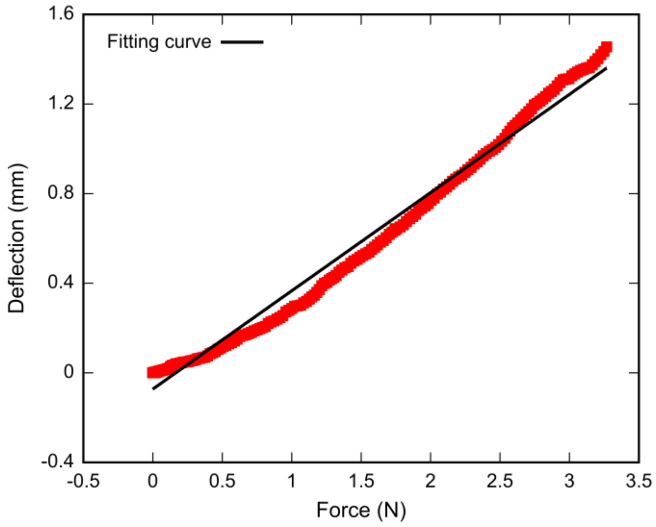

Supplementary Figure D5. Metallic spring with a rubber coating. Deflection vs. applied force characteristic, in the configuration of a beam clamped at both extremities subjected to both bending and force solicitations.

Supplementary Data D3

Three experimental setups were built to investigate the three typical mechanical configurations (i.e., (a) cantilever and (b) eccentrically loaded beam subjected to bending and buckling, respectively, and (c) beam clamped at both extremities subjected to both bending and force) of the soft sensing body. They are depicted in the following figures.

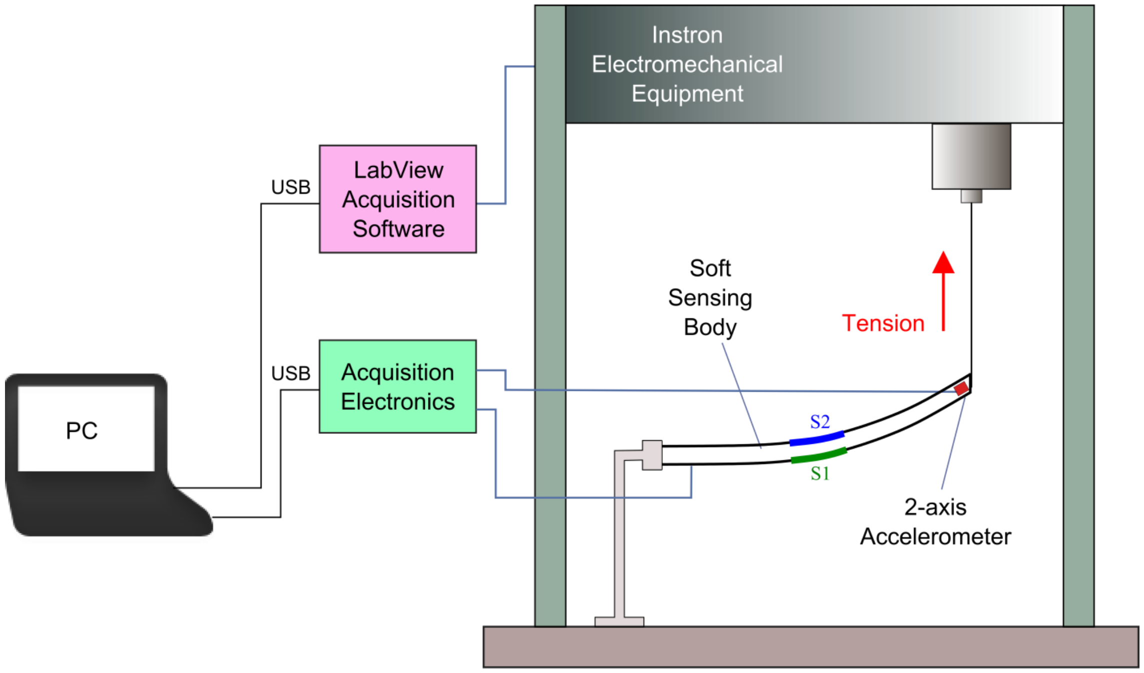

Supplementary Figure D6. The schematic depicts the experimental setup (not in scale) used for applying a bending stimulation to the soft sensing body in the cantilever beam configuration and for acquiring data during the characterization (the soft sensing body subjected to such solicitation is shown in Fig. D9a). S1 and S2 are the sensing elements positioned at the centre of the soft body, at the convex and concave sides, respectively. The results of this experiment are shown in Fig. 3b-c of this work.

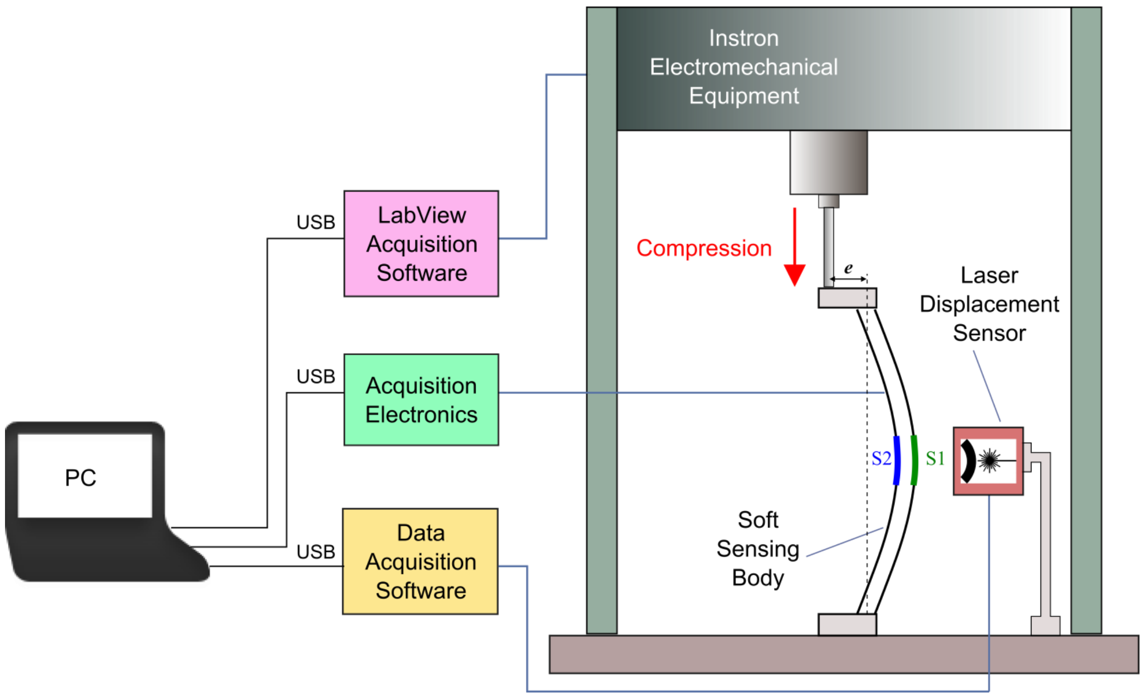

Supplementary Figure D7. The schematic represents the experimental setup (not in scale) for the characterization of the eccentrically loaded beam configuration (with eccentricity e between the beam vertical axis and the application point of the concentrated force F) when it is subjected to buckling (the sensing body in this configuration is depicted in Fig. D9b). S1 and S2 are the sensing elements positioned at the centre of the soft body, at the convex and concave sides, respectively. A laser displacement sensor is used to measure the beam maximum deflection. The results of this experiment are shown in Fig. 4b-c of the main text.

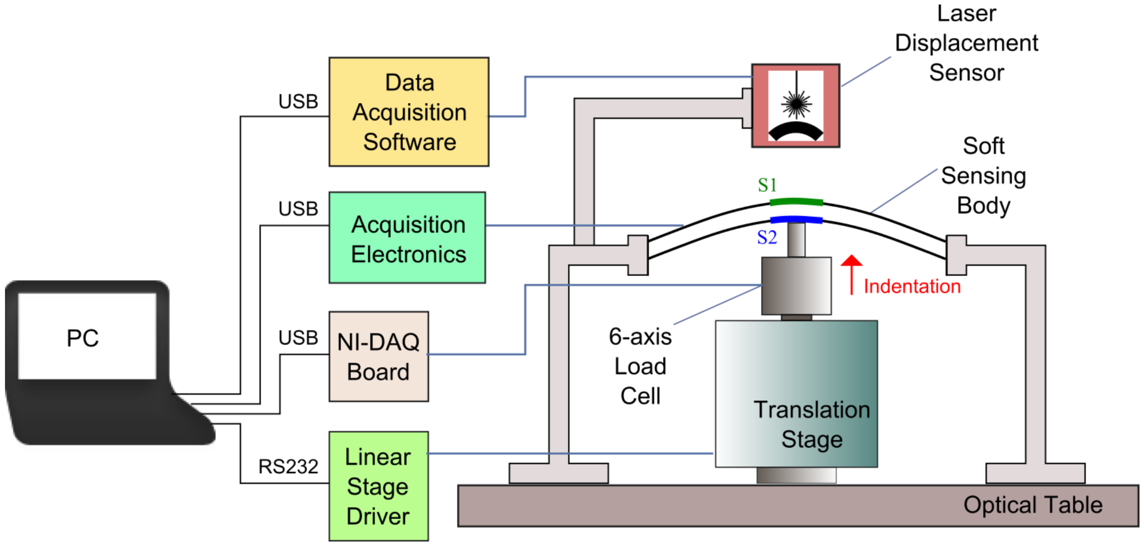

Supplementary Figure D8. The schematic shows the experimental setup (not in scale) for applying a bending solicitation by means of an external force to the soft sensing body clamped at both extremities, together with the acquisition system employed during the characterization (the sensing body in this configuration is shown in Fig. D9c). S1 and S2 are the sensing

elements positioned at the centre of the soft body, at the convex and concave sides, respectively. The results of this experiment are shown in Fig. 5b-e of this work.

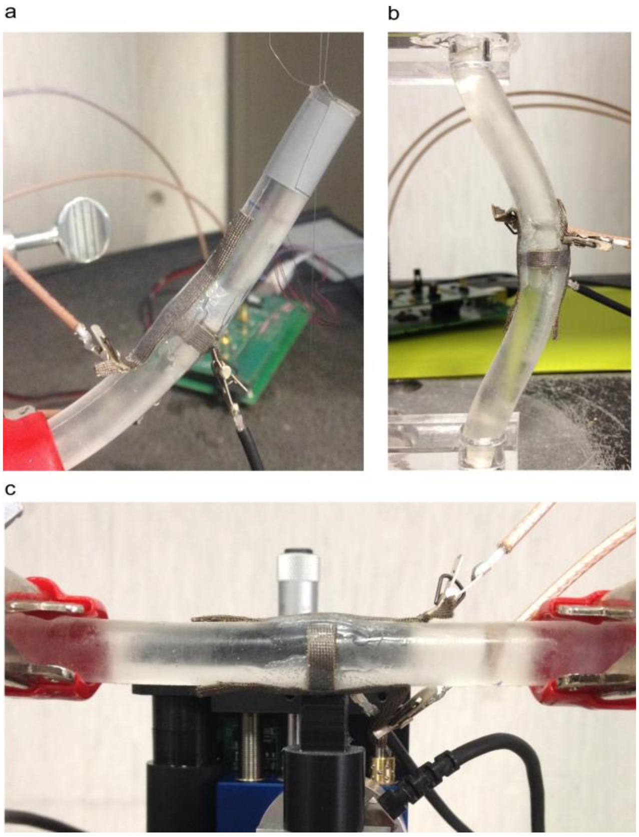

Supplementary Figure D9. Solicitations applied to the soft sensing body. (a) Illustration of the sensing body in the cantilever beam configuration with a concentrated force at the free end and the sensing elements S1 and S2 located at the beam centre. (b) Picture depicting the buckling of the soft sensing body with eccentricity between the beam vertical axis and the load application point, with the sensing elements S1 and S2 positioned at the centre of the beam. (c) Illustration of the sensing body clamped at both extremities with an external force applied at the middle and the sensing elements S1 and S2 positioned at the beam centre.

Supplementary Data D4

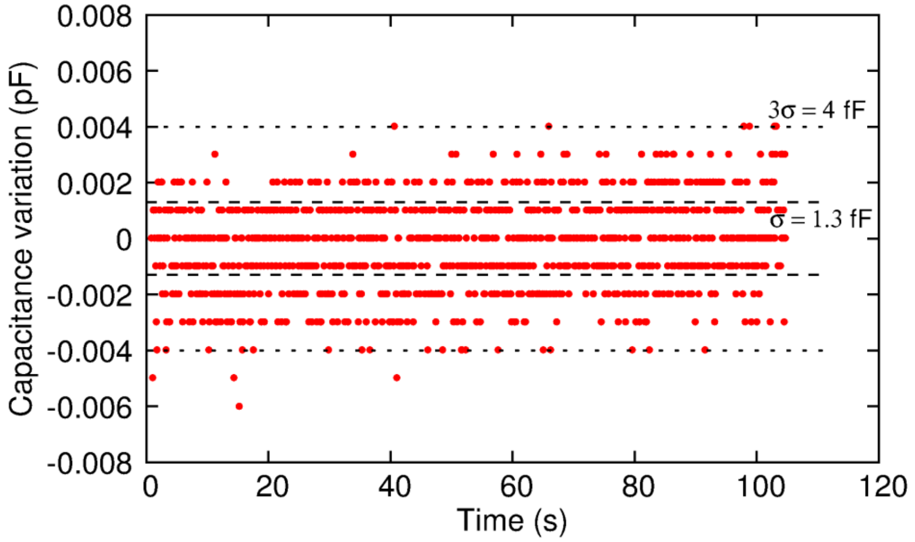

Supplementary Figure D10. In the graph the output noise is shown. In particular, the RMS noise is around 1.3 fF. Then, the minimum detectable signal (defined as three times the RMS value) is 4 fF. We can note the discretized levels of the signal, due to the CDC, whose resolution is 1 fF.
